# Cluster analysis of host cytokine responses to biodefense pathogens in a whole blood *ex vivo* exposure model (WEEM)

**DOI:** 10.1186/1471-2180-12-79

**Published:** 2012-05-20

**Authors:** Brett A Chromy, Imola K Fodor, Nancy K Montgomery, Paul A Luciw, Sandra L McCutchen-Maloney

**Affiliations:** 1 Physical and Life Sciences Directorate, Lawrence Livermore National Laboratory, 7000 East Avenue, Livermore, CA, 94550, USA; 2 Computation Directorate, Lawrence Livermore National Laboratory, 7000 East Avenue, Livermore, CA, 94550, USA; 3 Center for Comparative Medicine, University of California at Davis, Sacramento, CA, 95817, USA; 4 Department of Pathology and Laboratory Medicine, University of California Davis School of Medicine, Sacramento, CA, 95817, USA; 5 Current address: Department of Biostatistics, Genentech, Inc., 1 DNA Way, South San Francisco, CA, 94080, USA

## Abstract

**Background:**

Rapid detection and therapeutic intervention for infectious and emerging diseases is a major scientific goal in biodefense and public health. Toward this end, cytokine profiles in human blood were investigated using a human whole blood *ex vivo* exposure model, called WEEM.

**Results:**

Samples of whole blood from healthy volunteers were incubated with seven pathogens including *Yersinia pseudotuberculosis*, *Yersinia enterocolitica, Bacillus anthracis*, and multiple strains of *Yersinia pestis*, and multiplexed protein expression profiling was conducted on supernatants of these cultures with an antibody array to detect 30 cytokines simultaneously. Levels of 8 cytokines, IL-1α, IL-1β, IL-6, IL-8, IL-10, IP-10, MCP-1 and TNFα, were significantly up-regulated in plasma after bacterial exposures of 4 hours. Statistical clustering was applied to group the pathogens based on the host response protein expression profiles. The nearest phylogenetic neighbors clustered more closely than the more distant pathogens, and all seven pathogens were clearly differentiated from the unexposed control. In addition, the *Y. pestis* and *Yersinia* near neighbors were differentiated from the *B. anthracis* strains.

**Conclusions:**

Cluster analysis, based on host response cytokine profiles, indicates that distinct patterns of immunomodulatory proteins are induced by the different pathogen exposures and these patterns may enable further development into biomarkers for diagnosing pathogen exposure.

## Background

*Yersinia pestis* and *Bacillus anthracis* are two pathogens of significant concern to public health from a biodefense perspective [1,2]. *Y. pestis*, the causative agent of plague, is a Gram-negative, highly communicable coccobacillus that has been responsible for three historic pandemics with high mortality rates [3-5]. The microorganism possesses a Type III secretion mechanism common to several human, animal and plant pathogens, whereby a series of pathogen-specific structural proteins form a syringe-like structure capable of injecting virulence factors into the mammalian host cell. These virulence factors then facilitate pathogen use of host nutrients and thwart the host immune response, ultimately causing cell and host death [6,7]. Naturally occurring plague can be transmitted from infected fleas and rodents to humans, and although the pathogen can be phagocytosed, it can also resist destruction by manipulating the host defense mechanism(s), potentially through antigenic mimicry [8]. *Y. pestis* then multiplies rapidly leading to necrosis of lymph nodes, a condition known as bubonic plague, which can result in death if untreated [2]. In some cases the infection can spread through the blood stream resulting in systemic plague (septicemia) or to the lungs resulting in the highly contagious and deadly form of the disease known as pneumonic plague. There are currently no rapid, widely available diagnostic tests for plague, and the most common treatment is streptomycin [2,3], an antibiotic with adverse effects.

Two other species from the genus *Yersinia* are also human pathogens: *Y. pseudotuberculosis* and *Y. enterocolitica*[9,10]. Despite their high degree of sequence similarity to *Y. pestis*, these two near neighbors of *Y. pestis* manifest in very different symptoms, ranging from abdominal pain to septicemia in humans, usually caused by infection through contaminated food. Infections caused by *Y. pseudotuberculosis* or *Y. enterocolitica* can be effectively treated with antibiotics and in most cases are self-limiting. Notably, *Y. pestis* is reported to have evolved from *Y. pseudotuberculosis* within the past 10,000 years [11].

*B. anthracis* is a Gram-positive, rod-shaped spore-forming bacterial pathogen and the causative agent of anthrax [12,13]. Human, livestock, and wildlife mortalities attributable to anthrax occur in numerous regions of the world, although the majority of cases are found in less industrialized nations [14]. Three forms of the disease have been described: cutaneous, intestinal and inhalational. While cutaneous and intestinal forms may be less severe, inhalational anthrax is often fatal without prompt antibiotic treatment [13]. The primary mechanisms of virulence employed by *B. anthracis* are associated with two virulence plasmids designated pXO1 and pXO2 [15]. The net effect of these plasmids is virtually unhindered proliferation of *B. anthracis* within the host, hemorrhaging, cardio-pulmonary collapse, and death.

The regulation of production of host cytokines by both *Yersinia* and *B. anthracis* has been described previously. Pickering A. K. *et. al*. measured cytokine levels in human dendritic cell supernatant and in mouse peritoneal macrophages exposed to *B. anthracis* spores [16]. They observed significant increase in TNF-α, IL-6, IL-1β, IL-8, and IL-12 in human dendritic cell supernatants by 5 hours post-exposure. High levels of IL-6, and TNF-α were observed in the supernatant from *B. anthracis* infected mouse peritoneal macrophages [16]. In a mouse model, 6 cytokines, namely IL-12p70, TNF, IFN-γ, MCP-1, IL-10, and IL-6, were increased significantly in mouse lung at 48 hours of *Y. pestis* infection [17]. In previous work comparing exposures to different bacterial pathogens, distinct patterns of cytokine expression levels were found that could discriminate the particular host response [18], including while using pathogen-specific LPS in whole blood [19].

The hypothesis for the present study is that exposure to diverse bacterial pathogen strains would result in distinct cytokine profiles in the host, with strains from the same species exhibiting more similar profiles than strains from phylogenetically distant species. A multiplex cytokine protein chip was used, and a multivariate approach was taken that combined expression data on multiple cytokines. Multivariate clustering techniques were used to establish cytokine expression profiles after *ex vivo* exposure of whole blood to seven pathogens.

## Methods

### Bacterial strains and culture conditions

The bacterial strains used in this study include: *B. anthracis* Ames (virulent), *B. anthracis* Sterne (vaccine strain), *Y. pestis* KIM5 D27 (attenuated, pgm-). *Y. pestis* India/P (attenuated, pgm-), and *Y. pestis* NYC (virulent), *Y. pseudotuberculosis* serotype 1 PB1, and *Y. enterocolitica* WA serovar 0:8. Bacteria were grown on tryptose blood agar slants at 26°C for 1-2 days and subsequently collected using 2 ml of 0.033M potassium-phosphate, pH 7.0;.bacterial densities were measured at OD_620_ (1 OD_620_ = 1.2 *x* 10^9^ colony forming units/ml).

### Whole blood ex vivo exposure model (WEEM)

Human blood was collected from a healthy donor by venipuncture using CPT Vacutainer tubes (Becton Dickinson) containing citrate. Informed consent was obtained and our blood collection protocol was approved by the LLNL IRB committee. Separate CPT tubes were used for the unexposed control and 7 different bacterial exposures (*B. anthracis* Ames, *B. anthracis* Sterne, *Y. pestis* NYC, *Y. pestis* India/P, Y*. pestis* KIM5 D27, *Y. pseudotuberculosis*, and *Y. enterocolitica*). Bacteria were added to blood within 15 minutes of collection at a multiplicity of infection ratio of 5:1. This ratio was determined against white blood cells in whole blood:∼7 x 10^6^ cells/ml. Each whole blood sample was incubated with bacteria for 4 hours at 37°C in 5% CO_2_ Following incubation, plasma was collected by centrifugation at 2000 x g for 10 min at 4°C. The control plasma was obtained in the same way and treated with 0.033 M potassium-phosphate as a mock exposure. These plasma samples were used for cytokine measurements.

### Cytokine immunoassays with protein arrays

The measurements of cytokines were performed using Zyomyx Protein Profiling Biochips (Hayward, CA). These protein arrays allow the simultaneous quantification of 30 biologically relevant cytokines, as determined by Zyomyx, Inc: IL-1α, IL-1β, IL-2, IL-3, IL-4, IL-5, IL-6, IL-7, IL-8, IL-10, IL-12(p40), IL-12(p40/p70), IL-12(p70), IL-13, IL-15, TNFα, TNFβ, Eotaxin, MCP-1, MCP-3, TRAIL, CD95(sFas), MIG, sICAM-1, IP-10, CD23, TGF-β, GM-CSF, GCSF, IFN-γ. Each cytokine assay was optimized for the Zyomyx Protein Profiling Biochip based on many factors including the availability of antibodies and the sensitivity and specificity of antibody-cytokine interactions. Each protein array chip is designed with 6 independent microfluidic channels that allow up to 6 samples to be loaded into isolated regions of an array. Antibodies specific for 30 analytes were arrayed in each channel, and each antibody was arrayed in redundancy on 5 pillars within the channel. Accordingly, a cytokine measurement represents the average of 5 measurements. All immunoassay steps, including sample loading, washing, and detection, were performed with a fully automated biochip processing station (Zyomyx Assay 1200 workstation). Eight protein array chips were used in these experiments. Two chips were used for generating calibration curves with a calibration standard kit containing 30 analytes (Zyomyx, Inc.). Sample (40 μl) was injected into each channel of the protein array chips. Standard solutions were applied to two channels of each chip for chip-to-chip normalization. Triplicates of control and pathogen-exposed plasmas were applied randomly to four channels of 6 protein array chips. Protein arrays were scanned at 532 nm with Zyomyx Scanner 100 after immunoassays. Zyomyx Data Reduction software was used for normalization, calculation of calibration curves. Dixon’s test was used to remove outliers, and the median feature intensity was background subtracted. Concentrations of cytokines in plasma samples were determined by a four parameter logistic model.

### Cluster analysis of cytokine data

Multiple hierarchical clustering methods were used to group the pathogen exposures based on the multivariate cytokine expression profiles induced in a host infection model system. First, hierarchical agglomerative clustering [20] was applied to group the control and the seven pathogen-exposed samples based on their cytokine concentration profiles. Each of the eight samples was characterized by the multivariate vector of its average log10 concentration over the cytokines. The samples were then clustered based on the following distance measures between the samples and between the clusters.

Distance between two samples was defined using two distance metrics:

· Euclidean distance

· Correlation distance: (1 – Spearman correlation coefficient between the samples)

Distance between two clusters was defined using three methods:

· Complete linkage (furthest neighbor): the largest distance between members of the clusters

· Single linkage (nearest neighbor): the smallest distance between members of the clusters

· Average linkage (group average): the average distance between members of the clusters

Given a pair of distance metrics between samples and clusters, the algorithm was initialized with the eight samples forming eight different clusters and then processed iteratively by joining the two most similar clusters. The tree was built starting from the individual samples, using an agglomerative (bottom up) approach. The resulting hierarchy of clusters was displayed as a dendrogram. These traditional clustering methods provide a quick, exploratory overview of the data. However, these methods do not estimate the optimal number of clusters in the data; rather, the clustering is performed exhaustively from the lowest possible level of the hierarchy where each sample forms its own cluster, to the highest level where all samples are grouped into one cluster.

In addition to the traditional hierarchical agglomerative clustering method, the hierarchical ordered partitioning and collapsing hybrid (HOPACH) algorithm was also applied to the cytokine measurements [21]. In contrast with the previous approaches where the tree was built starting from the individual samples as the leaf nodes, HOPACH used a hybrid divisive-agglomerative approach: it started from the root cluster containing all the samples (divisive, top down approach), then divided the root down to leaf nodes, with an extra collapsing (agglomerative) step after each iteration that combined similar clusters. Based on the correlation distance between samples, HOPACH determined the split that minimized a measure of cluster homogeneity called the median split silhouette. While computationally more expensive than the previous methods, HOPACH was expected to perform better because of its dynamic approach to update and potentially revise the clusters at every step of the iteration. Furthermore, HOPACH also estimated the optimal number of clusters from the data, and thus offered another advantage over the previous methods.

Computations were performed in the R computing environment (http://www.r-project.org/) and the HOPACH package [21].

## Results

**Figure 1:**
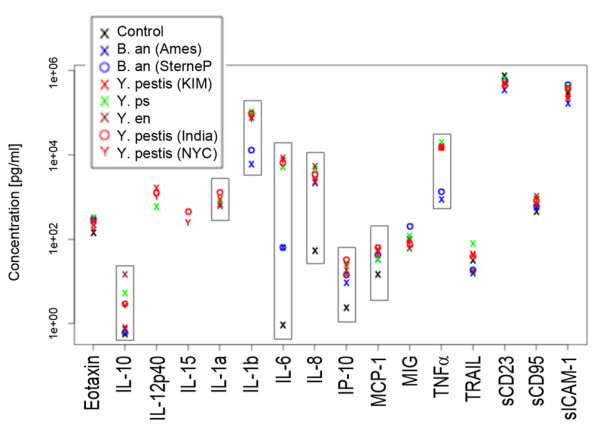
**Scatter plots of 16 cytokine concentrations detected in human blood following* ex vivo *bacterial exposures.** Cytokine concentrations were displayed on a logarithmic scale. The cytokines shown here were detected out of the 30 cytokines in the arrays. The 8 cytokines that were found to be statistically differentially expressed among these samples are highlighted with rectangular boxes. Each mark delineates the average of triplicate exposure samples. Each exposure sample is loaded onto a protein array chip that contains 5 independent measurements per cytokine meaning that fifteen measurements are used to obtain these data.

**Figure 2:**
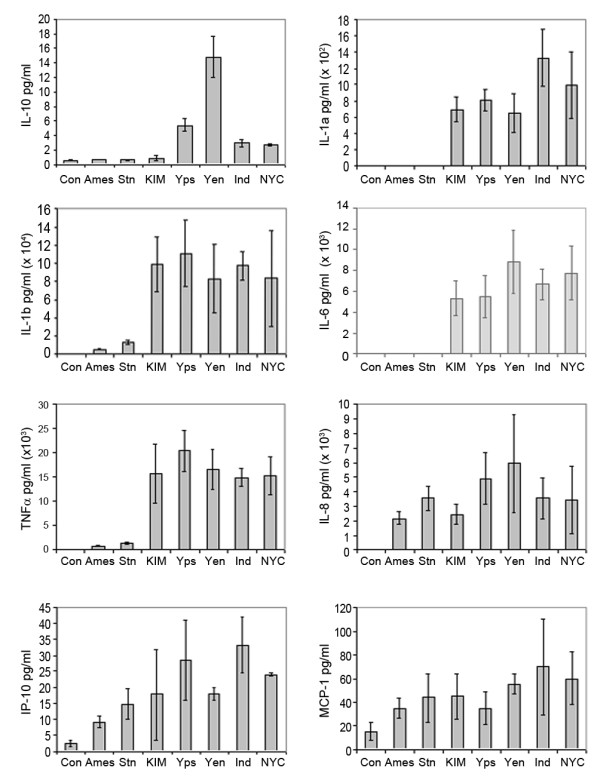
**Concentrations of 8 cytokines in human whole blood after* ex vivo *exposure to pathogens.** The control was a mock-exposed sample. Cytokine concentrations were determined using protein arrays. The bars represent the average of three replicate samples that each contain 5 replicate features per cytokine assay and the lines represent the standard deviation among the three replicates.

Cytokine levels were examined using an *ex vivo* model, termed WEEM for whole blood *x vivo*exposure model. Individual samples of anti-coagulated human blood were incubated with *B. anthracis* Ames, *B. anthracis* Sterne, *Y. pestis* KIM5 D27, *Y. pestis* NYC, *Y. pestis* India/P, *Y. enterocolitica*, and *Y. pseudotuberculosis*. After 4 hours exposure, blood cells were removed by low-speed centrifugation and concentrations of 30 cytokines in the plasma were measured with protein arrays. Concentrations of fourteen cytokines, GCSF, IFNγ, GM-CSF, IL-7, IL-12(p70), IL-12(p40/p70), IL-13, IL-2, IL-3, IL-4, IL-5, MCP-3, TGFβ, and TNFβ were below the limit of detection in this study. The following 16 cytokines were detected: Eotaxin, IL-10, IL-12(p40), IL-15, IL-1α, IL-1β, IL-6, IL-8, IP-10, MCP-1, MIG, TNFα, TRAIL, sCD23, sCD95, and sICAM-1 (Figure 1). To determine if there were significant differences among the levels of cytokines in the control and pathogen exposed plasma samples, F-tests were performed. For thirteen of these 16 cytokines, all three replicates were detected and these cytokines were subjected to F-tests. Statistical analysis indicated that 8 cytokines (IL-1α, IL-1β, IL-6, IL-8, IL-10, IP-10, MCP-1, and TNFα) had differentially elevated expression profiles following different bacterial exposures. Figure 2 shows the concentrations (pg/ml) of these cytokines in the control and bacteria exposed plasma samples. The F-tests revealed that the other five cytokines containing complete datasets, TRAIL, sCD23, sCD95, MIG, and sICAM-1, had no significant difference between bacterial exposures and the mock-exposed control. Moreover, there was a great variation in absolute concentrations between cytokines. For example, the concentrations of TNFα, sCD23, and sICAM-1 were as high as 1 x 10^4^ -10^5^ pg/ml, whereas IL-10 was much lower, about 16 pg/ml.

Marked differences in induced cytokine patterns between *B. anthracis* and *Yersinia* exposures were found. Also, the levels of induction of these cytokines differed among the different bacteria. For example, *Yersinia* species induced much higher cytokine response than *B. anthracis* for IL-1α, IL-1β, IL-6, and TNF-α (Figure 2). The two strains of *B. anthracis* bacteria induced different levels of IL-1β and TNF-α (Figure 2), including 2.2 times higher concentration of IL-1β and 1.6 times higher concentration of TNF-α for the Sterne strain than the Ames strain of *B. anthracis*. These differences were statistically significant (pairwise *t*-test p value = 0.0039 for IL-1β and 0.022 for TNF-α). To discriminate *Y. pestis* exposure from near neighbors, IL-10 levels can be used, showing cytokine concentrations following *Y. enterocolitica* exposure and *Y. pseudotuberculosis* exposure that are on average 5-fold higher and 2-fold higher, respectively, than after *Y. pestis* exposure (Figure 2). IL-10 differential expression was specific to the *Yersinia spp*. because exposure to *B. anthracis* strains showed comparable IL-10 levels to that in unexposed control.

**Figure 3:**
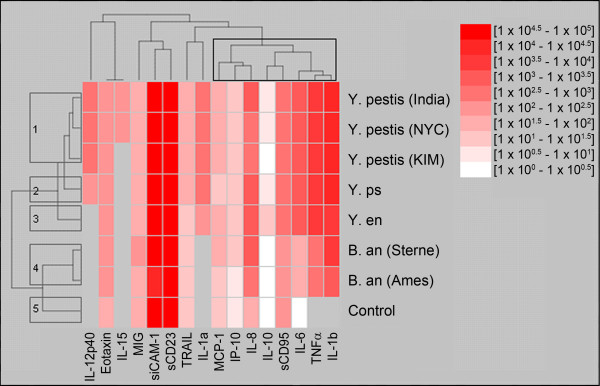
**Clustering result with HOPACH using the average linkage distance between clusters is shown.** The eight pathogen-exposed samples are clustered according to the dendrogram on the left and cluster into five groups, 1) *Y. pestis* (KIM5, NYC, and India), 2) *Y. pseudotuberculosis*, 3) *Y. enterocolitica*, 4) *B. anthracis* (Ames and Sterne), and 5) Control. Sixteen cytokines (Eotaxin, IL-10, IL-12(p40), IL-15, IL-1α, IL-1β, IL-6, IL-8, IP-10, MCP-1, MIG, TNFα, TRAIL, sCD23, sCD95, and sICAM-1) are also reordered based on their correlations according to the dendrogram on the top. Clusters go from root at top to leaf node for each cytokine. Clusters in between are based on their agglomerative . The branch shows the similarity, the short the branch, the more similar. In addition, the eight rightmost proteins form a cluster that may involve inflammation-related cascades initiated by an innate immune response to these pathogen. Colors represent units of log10 [pg/ml], in ten equally spaced intervals increasing from white to dark red. A key showing the specific log10 values for each interval is shown in the figure.

The HOPACH algorithm estimated the number of clusters as five, and grouped the samples based on their host cytokine expression profiles as follows: 1) *Y. pestis* (KIM5 D27, India/P, and NYC), 2) *Y. pseudotuberculosis, 3) Y. enterocolitica, 4) B. anthracis* (Ames and Sterne), and 5) Control (Figure 3). The closer the pathogen-exposed samples are within the tree on the left, the more similar they are. Height of the branches indicates the distance between the successive nodes in the clustering. The method separated the *B. anthracis* and *Yersinia* infected blood samples. In addition, the cytokine profile of the mock-exposed control was more similar to the pattern produced by *B. anthracis* exposure than to the profile elicited by *Yersinia*.

Results of the hierarchical clustering when using the Euclidean distance between samples depended on the distance metric between clusters. The three methods for determining the distance between clusters (complete linkage, single linkage, and average linkage, see Materials and Methods) all established three major clusters: 1) *Y. pestis* and near neighbors, 2) *B. anthracis*, and c) Control. Differences between the results occurred when the *Yersinia* cluster was further divided. The average linkage method, consistent with Figure 3, formed a subgroup of the three *Y. pestis* strains, then grouped them first with *Y. pseudotuberculosis* followed by *Y. enterocolitica.* Complete and single linkage methods, however, first grouped the attenuated virulent strain of *Y. pestis* (India/P) with the more virulent strain (NYC)*,* both clinical isolates from human plague cases, and then clustered them with *Y. pseudotuberculosis*, followed by the attenuated *Y. pestis* (KIM5 D27)*,* and lastly with *Y. enterocolitica*. This is interesting from an evolutionary perspective because it has been proposed that *Y. pestis* evolved from *Y. pseudotuberculosis* within the last 10,000 years, and thus these two pathogens are more closely related [11].

When using hierarchical clustering with the correlation distance between the samples, the final clusters were independent of the distance metric between clusters, and agreed with the tree structure in Figure 3. The complete, single, and average linkage methods all resulted in the following major clusters: 1) *Yersinia*, 2) *B. anthracis*, and 3). Control. Within the *Yersinia* cluster, *Y. pestis* (NYC) was closest to *Y. pestis* (India/P), followed by *Y. pestis* (KIM5 D27), *Y. pseudotuberculosis*, and *Y. enterocolitica*.

## Discussion

The HOPACH clustering method (Figure 3) produced five distinctly separated clusters: 1) *Y. pestis* (KIM5 D27, India/P, and NYC), 2) *Y. pseudotuberculosis, 3) Y. enterocolitica, 4) B. anthracis* (Ames and Sterne), and 5) Control. This result is consistent with the findings using the correlation distance and the Euclidean distance with average linkage. In addition, HOPACH estimated the optimal number of clusters as five. That is, the *Yersinia* subcluster is best if it is divided into the three clusters specified by 1) through 5) above. *Y. enterocolitica* forms its own cluster, and so does *Y. pseudotuberculosis*. *Y. pestis* (KIM5 D27), *Y. pestis* (India/P), and *Y. pestis* (NYC) are grouped into one cluster. Further subdivisions lead to an overall clustering with inferior quality.

In addition to clustering the cytokine expression profiles across bacterial treatments, Figure 3 also groups the cytokines themselves and clusters the proteins based on their similarities across the pathogen exposures and reorders them accordingly. Interestingly, the three pro-inflammatory cytokines IL-1β, TNFα, and IL-6 clustered closely, and so did the three chemokines MCP-1, IP-10, and IL-8. Although these 6 cytokines do not cluster as a single group, they do cluster at a branch further away from the leaf node, which includes IL-10 and sCD95, to make a larger group of 8 proteins. Several of these proteins are involved in inflammatory conditions, such as IL-1beta, TNFα, IL-6, [22] and have been shown to be upregulated in cell culture and animal model specifically exposed to biothreat agents [23]. Increased expression of IL-6 and TNFα clustered together in a study involving mouse splenic CD11b + cells following sub-lethal *Y. enterocolitica* infection [24]. In addition, several of the cytokines in this cluster, namely TNF-alpha, IL1-beta, IL-10, and MCP-1 are expressed higher in exposed whole blood as compared to control in this study and in whole blood exposure to LPS from several other gram negative bacterial pathogens [19]. In addition to expression differences, the absence of detected cytokine expression can also be helpful in discriminating pathogen exposure.

The multiplex detection of 30 cytokines in this study revealed the early phase cytokine expression profiles in human plasma following exposures to *B. anthracis* (Ames and Sterne), *Y. pestis* (KIM5 D27, NYC and India/P), *Y. pseudotuberculosis*, and *Y. enterocolitica*. The expression levels of 8 cytokines, IL-1α, IL-1β, IL-6, IL-8, IL-10, IP-10, MCP-1, and TNFα were significantly different from that of unexposed control (Figure 2). Although the focus of our work was to show that cytokine expression profiling can discriminate between different pathogen exposures in a human whole blood *ex vivo* model, these results also represent an initial attempt to characterize the full cytokine response to each individual pathogen. Our preliminary study using a single exposure protocol at a single time post-exposure will need to be supplemented with more thorough investigation in order to determine the usefulness of using cytokine levels for diagnosing pathogen exposure. However, the single time point chosen, 4 hours, is sufficient to detect proteomic changes and has been used in previous studies examining cytokine levels [25-27]. This time point represents a start towards a more complete temporal study, as has been done with gene expression patterns for two of the pathogens studied here [25,27]. In addition, studies that provide expression patterns for a single cytokine using multiple time points will also be needed to make the results of this paper clinically useful, such as has been done by, Cooper and coworkers, who examined IL-12p40 and IL-12p70 levels following different growth conditions and exposure levels for a time course of *Y. pestis* exposed dendritic cells [28]. The results of the current work shows a similar expression pattern trend to this previous work, in which, *Y. pestis* induces IL-12p40 and at a substantially higher level than IL-12p70.

Our results showed that the expression levels of 3 chemokines, IL-8, MCP-1 and IP-10, were induced by both *Yersinia* and *B. anthracis* exposures. No significant differences were found for these cytokines between *Yersinia* and *B. anthracis* exposures. IL-8, MCP-1 and IP-10 are chemokines that enable the migration of leukocytes from the blood to the site of inflammation. IL-8 is a key chemokine regulating neutrophil recruitment [29]. The essential involvement of IL-8 in acute inflammation was demonstrated by neutralizing IL-8 with its antibody. When highly specific antibody against IL-8 was administered in acute inflammatory reactions induced by several stimuli including lipopolysaccharide, neutrophil infiltration was blocked [30]. MCP-1 is known for its ability to act as potent chemoattractant and activator of monocytes/macrophages as well as NK cells but not neutrophils [31,32] . IP-10 has no chemotactic activity for neutrophils but attracts monocytes, NK, and T cells to the site of infection and regulates T cell maturation [33,34]. It was reported previously that elevated IL-8 and MCP-1 were secreted by human epithelial cells after *Y. enterocolitica* infection, but not IP-10 [35,36]. Human dendritic cells, infected with *B. anthracis* spores, secreted high level of IL-8 at 7.5 hours [16]. In our study, the fold increase of IL-8 was much greater than MCP-1 and IP-10 (Figure 2). For example, the induction of IL-8 by Ames strain of *B. anthracis* was 41 fold, while MCP-1 was 2 fold and IP-10 was 2.5 fold (Figure 2). This result may indicate that IL-8 is a dominant chemokine in early response (4 hours exposure in our study) and neutrophils are the major player in early inflammatory response.

Here we compared cytokines induced by *B. anthracis* and *Yersinia* exposures. Overall, *Yersinia* exposure induced higher levels of IL-1α, IL-1β, IL-6, IL-10 and TNFα than *B. anthracis* exposure, suggesting these cytokines could be used to develop an assay for discriminating *Yersinia spp.* from *B. anthracis* exposures. The vaccine strain (Sterne) of *B. anthracis* induced higher levels of IL-1β and TNFα than the virulent strain (Ames) (Figure 2), suggesting these cytokines can contribute to a biomarker panel to discriminate if a particular isolate of *B. anthracis* is virulent. There was also a difference in induction of IL-10 between *Y. pestis* and near neighbors (Figure 2), suggesting this cytokine is a candidate biomarker for discriminating the virulence of *Yersinia* species. These data regarding IL-10 expression following *Yersinia spp.* exposure are in agreement with published literature that shows *Y. enterocolitica* and *Y. pestis* can elicit statistically different levels of IL-10 expression [37]. Differences in IL-10 induction may be due to differences in the lcrV protein among *Yersinia spp.*[38]. The different cytokine profiles induced by *B. anthracis* and *Yersinia* here may be partially due to different surface antigens on the outermost part of these pathogens and the manner in which these bacteria were grown. Lipopolysaccharide (LPS), the main constituent of the outer membrane of Gram-negative bacteria, and peptidoglycan (PGN), the major cell wall component of Gram-positive bacteria, have been reported to elicit markedly different immune responses [39]. However, virulence factors, such as *B*. *anthracis* lethal toxin and *Yersinia* virulence antigen, LcrV, may also play important roles in differential cytokine induction. This view is supported by numerous reports that *B. anthracis* toxin and virulence factors of *Yersinia* bacteria (Yops, invasin, LcrV) modulate host cytokine responses [40-51].

While the various clustering methods resulted in slightly different final hierarchies, all were consistent in separating the unexposed control from the samples exposed to *B. anthracis* or to the *Y. pestis* and near neighbors. Agreement on this level among the various clustering procedures lends more confidence to the overall results. On a more detailed level, the methods grouped slightly differently the samples exposed to the *Y. pestis* and near neighbors, which indicates that these samples cannot be unequivocally separated based on the current data and additional biomarkers or a larger sample set would be needed. The most advanced HOPACH method estimated the optimal number of clusters in the data as five, corresponding to the unexposed control, and the four species: *B. anthracis*, *Y. pseudotuberculosis, Y. enterocolitica,* and *Y. pestis* (avirulent and virulent) (Figure 3)*.*

Information gained from the targeted protein array data for host response complements genomic [52-56], and other proteomic studies [57-60] of host-pathogen interactions. The success of the WEEM and computational method to distinguish pathogen exposure, based on host response in this initial study, is encouraging and suggests a number of possibilities for future studies to refine the findings. Comparative analysis, such as the current work, can potentially reveal the critical pathogenic mechanism(s) and host innate immune responses during infection as was previously shown for *Y. pestis* and *Y. pseudotuberculosis*[61]. Opportunities include using statistical hypothesis tests based on analysis of variance to assess the significance of the observed differences among the host-pathogen cytokine concentration profiles, as well as performing follow-up studies to focus more on the *Y. pestis* and near neighbor cluster. In addition, the methods can be extended to investigate host responses to diverse pathogens in multiple host model systems to cross validate the significance of the biomarkers to distinguish pathogen exposures.

## Conclusion

Results from this study suggest that cytokine arrays coupled with statistical clustering methods can distinguish exposures to pathogens, including multiple strains of *Y. pestis, Y. pseudotuberculosis, Y. enterocolitica,* and *B. anthracis*. These methods differentiate both near neighbors and distant evolutionary microbes based on host response data. The distinct cytokine profiles also provide insight into both the host response and virulence mechanisms of diverse pathogens. In summary, characterization of host responses based on cytokine profiles has translational application, potentially providing the identification of infectious diseases and leading toward the ultimate goal of presymptomatic detection *via* sentinel surveillance of pathogen exposure and appropriate treatment.

## Competing interests

The authors declare that they have no competing interests.

## Authors’ contributions

BC participated in the design of the study, conducted experiments, and drafted and finalized the manuscript. KM conducted experiments. IF participated in the design of the study, performed the statistical analysis, and helped draft the manuscript. PL helped draft the manuscript. SM conceived of the study, obtained funding, and participated in its design and coordination and helped to draft the manuscript. All authors read and approved the final manuscript.

## References

[B1] Bossi P Bricaire F Bioterrorism: management of major biological agents Cell Mol Life Sci 2006 63 2196 2212 10.1007/s00018-006-6308-z 16964582 PMC11136302

[B2] Inglesby TV Plague as a biological weapon: medical and public health management, Working Group on Civilian Biodefense JAMA 2000 283 2281 2290 10.1001/jama.283.17.2281 10807389

[B3] Stenseth NC Plague: past, present, and future PLoS Med 2008 5 e3 18198939 10.1371/journal.pmed.0050003 PMC2194748

[B4] Lee VT Schneewind O Protein secretion and the pathogenesis of bacterial infections Genes Dev 2001 15 1725 1752 10.1101/gad.896801 11459823

[B5] Perry RD Fetherston JD Yersinia pestis–etiologic agent of plague Clin Microbiol Rev 1997 10 35 66 8993858 10.1128/cmr.10.1.35 PMC172914

[B6] Matsumoto H Young GM Translocated effectors of Yersinia Curr Opin Microbiol 2009 12 94 100 10.1016/j.mib.2008.12.005 19185531 PMC2669664

[B7] Cornelis GR Yersinia type III secretion: send in the effectors J Cell Biol 2002 158 401 408 10.1083/jcb.200205077 12163464 PMC2173816

[B8] Stebbins CE Galan JE Structural mimicry in bacterial virulence Nature 2001 412 701 705 10.1038/35089000 11507631

[B9] Kutyrev V Expression of the plague plasminogen activator in Yersinia pseudotuberculosis and Escherichia coli Infect Immun 1999 67 1359 1367 10024583 10.1128/iai.67.3.1359-1367.1999 PMC96469

[B10] Cornelis GR The Yersinia Ysc-Yop 'type III' weaponry Nat Rev Mol Cell Biol 2002 3 742 752 10.1038/nrm932 12360191

[B11] Achtman M Yersinia pestis, the cause of plague, is a recently emerged clone of Yersinia pseudotuberculosis Proc Natl Acad Sci U S A 1999 96 14043 14048 10.1073/pnas.96.24.14043 10570195 PMC24187

[B12] Turnbull PC Introduction: anthrax history, disease and ecology Curr Top Microbiol Immunol 2002 271 1 19 12224519 10.1007/978-3-662-05767-4_1

[B13] Passalacqua KD Bergman NH Bacillus anthracis: interactions with the host and establishment of inhalational anthrax Future Microbiol 2006 1 397 415 10.2217/17460913.1.4.397 17661631

[B14] Hugh-Jones M 1996-97 Global Anthrax Report J Appl Microbiol 1999 87 189 191 10.1046/j.1365-2672.1999.00867.x 10475945

[B15] Kaspar RL Robertson DL Purification and physical analysis of Bacillus anthracis plasmids pXO1 and pXO2 Biochem Biophys Res Commun 1987 149 362 368 10.1016/0006-291X(87)90375-5 3122734

[B16] Pickering AK Cytokine response to infection with Bacillus anthracis spores Infect Immun 2004 72 6382 6389 10.1128/IAI.72.11.6382-6389.2004 15501768 PMC523056

[B17] Lathem WW Progression of primary pneumonic plague: a mouse model of infection, pathology, and bacterial transcriptional activity Proc Natl Acad Sci U S A 2005 102 17786 17791 10.1073/pnas.0506840102 16306265 PMC1308902

[B18] Cross ML Patterns of cytokine induction by gram-positive and gram-negative probiotic bacteriaFEMS Immunol Med 2004 42 2 173 180 10.1016/j.femsim.2004.04.001 15364101

[B19] Mathiak G Lipopolysaccharides from different bacterial sources elicit disparate cytokine responses in whole blood assays Int J Mol Med 2003 11 1 41 44 12469215

[B20] Kaufman L Rousseeuw PJ Finding Groups in Data: An Introduction to Cluster Analysis 1990

[B21] van der Laan MJ Pollard KJS Hybrid clustering of gene expression data with visualization and the bootstrap J Stat Planning and Inference 2003 117 275 303 10.1016/S0378-3758(02)00388-9

[B22] Ishikawa F Miyazaki S New biodefense strategies by neutrophils Arch Immunol Ther Exp (Warsz) 2005 53 3) 226 233 15995583

[B23] Das R Early indicators of exposure to biological threat agents using host gene profiles in peripheral blood mononuclear cells BMC Infect Dis 2008 30 8 104 10.1186/1471-2334-8-104 PMC2542375 18667072

[B24] Matteoli Role of IFN-gamma and IL-6 in a protective immune response toYersinia enterocoliticain mice BMC microbial 2008 8 153 10.1186/1471-2180-8-153 PMC2556677 18803824

[B25] Das R Study of proinflammatory responses induced by Yersinia pestis in human monocytes using cDNA arrays Genes Immun 2007 8 4 308 319 10.1038/sj.gene.6364389 17429414

[B26] Julkunen I Inflammatory responses in influenza A virus infection Vaccine 2000 8 19 Suppl 1 S32 S37 11163460 10.1016/s0264-410x(00)00275-9

[B27] Auerbuch V Golenbock DT Isberg RR Innate immune recognition of Yersinia pseudotuberculosis type III secretionDec PLoS Pathog 2009 5 12 e1000686 Epub 2009 Dec 4 10.1371/journal.ppat.1000686 19997504 PMC2779593

[B28] Robinson RT Yersinia pestisEvades TLR4-dependent Induction of IL-12(p40)2 by Dendritic Cells and Subsequent Cell MigrationJ Immunology 2008 181 5560 5567 10.4049/jimmunol.181.8.5560 PMC2640496 18832714

[B29] Singer M Sansonetti PJ IL-8 is a key chemokine regulating neutrophil recruitment in a new mouse model of Shigella-induced colitis J Immunol 2004 173 4197 4206 15356171 10.4049/jimmunol.173.6.4197

[B30] Harada A Essential involvement of interleukin-8 (IL-8) in acute inflammation J Leukoc Biol 1994 56 559 564 7964163

[B31] Morrison BE Park SJ Mooney JM Mehrad B Chemokine-mediated recruitment of NK cells is a critical host defense mechanism in invasive aspergillosis J Clin Invest 2003 112 1862 1870 14679181 10.1172/JCI18125 PMC296992

[B32] Baggiolini M Dewald B Moser B Human chemokines: an update Annu Rev Immunol 1997 15 675 705 10.1146/annurev.immunol.15.1.675 9143704

[B33] Christen U Cure of prediabetic mice by viral infections involves lymphocyte recruitment along an IP-10 gradient J Clin Invest 2004 113 74 84 14702111 10.1172/JCI200417005 PMC300760

[B34] Dufour JH IFN-gamma-inducible protein 10 (IP-10; CXCL10)-deficient mice reveal a role for IP-10 in effector T cell generation and trafficking J Immunol 2002 168 3195 3204 11907072 10.4049/jimmunol.168.7.3195

[B35] Kampik D Schulte R Autenrieth IB Yersinia enterocolitica invasin protein triggers differential production of interleukin-1, interleukin-8, monocyte chemoattractant protein 1, granulocyte-macrophage colony-stimulating factor, and tumor necrosis factor alpha in epithelial cells: implications for understanding the early cytokine network in Yersinia infections Infect Immun 2000 68 2484 2492 10.1128/IAI.68.5.2484-2492.2000 10768935 PMC97450

[B36] Jung HC Eckmann L Yang SK Panja A Fierer J Morzycka-Wroblewska E Kagnoff MF A distinct array of proinflammatory cytokines is expressed in human colon epithelial cells in response to bacterial invasion J Clin Invest 1995 95 55 65 10.1172/JCI117676 7814646 PMC295369

[B37] Reithmeier-Rost D The weak interaction of LcrV and TLR2 does not contribute to the virulence of Yersinia pestis Microbes Infect 2007 9 8 997 1002 10.1016/j.micinf.2007.04.003 17556003

[B38] Anisimov AP Variability of the protein sequences of lcrV between epidemic and atypical rhamnose-positive strains of Yersinia pestis Adv Exp Med Biol 2007 603 23 27 10.1007/978-0-387-72124-8_3 17966402

[B39] Van Amersfoort ES Van Berkel TJ Kuiper J Receptors, mediators, and mechanisms involved in bacterial sepsis and septic shock Clin Microbiol Rev 2003 16 379 414 10.1128/CMR.16.3.379-414.2003 12857774 PMC164216

[B40] Erwin JL Macrophage-derived cell lines do not express proinflammatory cytokines after exposure to Bacillus anthracis lethal toxin Infect Immun 2001 69 1175 1177 10.1128/IAI.69.2.1175-1177.2001 11160016 PMC98000

[B41] Hoover DL Anthrax edema toxin differentially regulates lipopolysaccharide-induced monocyte production of tumor necrosis factor alpha and interleukin-6 by increasing intracellular cyclic AMP Infect Immun 1994 62 4432 4439 7927706 10.1128/iai.62.10.4432-4439.1994 PMC303127

[B42] Arnold R Scheffer J Konig B Konig W Effects of Listeria monocytogenes and Yersinia enterocolitica on cytokine gene expression and release from human polymorphonuclear granulocytes and epithelial (HEp-2) cells Infect Immun 1993 61 2545 2552 8500890 10.1128/iai.61.6.2545-2552.1993 PMC280882

[B43] Brubaker RR Interleukin-10 and inhibition of innate immunity to Yersiniae: roles of Yops and LcrV (V antigen) Infect Immun 2003 71 3673 3681 10.1128/IAI.71.7.3673-3681.2003 12819047 PMC162007

[B44] Tournier JN Anthrax edema toxin cooperates with lethal toxin to impair cytokine secretion during infection of dendritic cells J Immunol 2005 174 4934 4941 15814721 10.4049/jimmunol.174.8.4934

[B45] Pellizzari R Anthrax lethal factor cleaves MKK3 in macrophages and inhibits the LPS/IFNgamma-induced release of NO and TNFalpha FEBS Lett 1999 462 199 204 10.1016/S0014-5793(99)01502-1 10580119

[B46] Grassl GA Activation of NF-kappaB and IL-8 by Yersinia enterocolitica invasin protein is conferred by engagement of Rac1 and MAP kinase cascades Cell Microbiol 2003 5 957 971 10.1046/j.1462-5822.2003.00339.x 14641180

[B47] Schulte R Yersinia enterocolitica invasin protein triggers IL-8 production in epithelial cells via activation of Rel p65-p65 homodimers FASEB J 2000 14 1471 1484 10.1096/fj.14.11.1471 10928981

[B48] Monnazzi LG Carlos IZ de Medeiros BM Influence of Yersinia pseudotuberculosis outer proteins (Yops) on interleukin-12, tumor necrosis factor alpha and nitric oxide production by peritoneal macrophages Immunol Lett 2004 94 91 98 10.1016/j.imlet.2004.04.007 15234540

[B49] Auerbuch V Golenbock DT Isberg RR Innate immune recognition of Yersinia pseudotuberculosis type III secretion PLoS Pathog 2009 5 e1000686 10.1371/journal.ppat.1000686 19997504 PMC2779593

[B50] Bergsbaken T Cookson BT Macrophage activation redirects yersinia-infected host cell death from apoptosis to caspase-1-dependent pyroptosis PLoS Pathog 2007 3 e161 10.1371/journal.ppat.0030161 17983266 PMC2048529

[B51] Shin H Cornelis GR Type III secretion translocation pores of Yersinia enterocolitica trigger maturation and release of pro-inflammatory IL-1beta Cell Microbiol 2007 9 2893 2902 10.1111/j.1462-5822.2007.01004.x 17991047

[B52] Achtman M Microevolution and history of the plague bacillus, Yersinia pestis Proc Natl Acad Sci U S A 2004 101 17837 17842 10.1073/pnas.0408026101 15598742 PMC535704

[B53] Huang XZ Nikolich MP Lindler LE Current trends in plague research: from genomics to virulence Clin Med Res 2006 4 189 199 10.3121/cmr.4.3.189 16988099 PMC1570480

[B54] Zhou D Han Y Song Y Huang P Yang R Comparative and evolutionary genomics of Yersinia pestis Microbes Infect 2004 6 1226 1234 10.1016/j.micinf.2004.08.002 15488743

[B55] Hinchliffe SJ Application of DNA microarrays to study the evolutionary genomics of Yersinia pestis and Yersinia pseudotuberculosis Genome Res 2003 13 2018 2029 10.1101/gr.1507303 12952873 PMC403674

[B56] Le Fleche P A tandem repeats database for bacterial genomes: application to the genotyping of Yersinia pestis and Bacillus anthracis BMC Microbiol 2001 1 2 10.1186/1471-2180-1-2 11299044 PMC31411

[B57] Chromy BA Proteomic characterization of host response to Yersinia pestis and near neighbors Biochem Biophys Res Commun 2004 320 474 479 10.1016/j.bbrc.2004.05.199 15219853

[B58] Zhang CG Chromy BA McCutchen-Maloney SL Host-pathogen interactions: a proteomic view Expert Rev Proteomics 2005 2 187 202 10.1586/14789450.2.2.187 15892564

[B59] Zhang CG Subcellular proteomic analysis of host-pathogen interactions using human monocytes exposed to Yersinia pestis and Yersinia pseudotuberculosis Proteomics 2005 5 1877 1888 10.1002/pmic.200401083 15825148

[B60] Sapra R Proteomic analyses of murine macrophages treated with Bacillus anthracis lethal toxin Microb Pathog 2006 41 157 167 10.1016/j.micpath.2006.07.002 16950595

[B61] Bergsbaken T Cookson BT Innate immune response during Yersinia infection: critical modulation of cell death mechanisms through phagocyte activation J Leukoc Biol 2009 86 1153 1158 10.1189/jlb.0309146 19734471 PMC2774879

